# In Vitro antibiotic combinations of Colistin, Meropenem, Amikacin, and Amoxicillin/clavulanate against multidrug-resistant *Klebsiella pneumonia* isolated from patients with ventilator-associated pneumonia

**DOI:** 10.1186/s12866-023-03039-w

**Published:** 2023-10-20

**Authors:** Ghazal Bayatinejad, Mohammadreza Salehi, Reza Beigverdi, Shahnaz Halimi, Mohammad Emaneini, Fereshteh Jabalameli

**Affiliations:** 1 Department of Microbiology, School of Medicine, Tehran University of Medical Sciences, Tehran, Iran; 2 Department of Infectious Diseases and Tropical Medicine, Imam Khomeini Hospital Complex, Tehran University of Medical Sciences, Tehran, Iran; 3 Research Center for Antibiotic Stewardship and Antimicrobial Resistance, Tehran University of Medical Sciences, Tehran, Iran

**Keywords:** Combination therapy, Synergism, Synergistic effect, Colistin, Carbapenem-resistant *Klebsiella pneumoniae*, Multidrug resistant pathogen, *Klebsiella pneumoniae*, Biofilm, Checkerboard method, Fractional inhibitory concentration (FIC).

## Abstract

**Background:**

Hospital infections such as ventilator-associated pneumonia (VAP) due to multidrug-resistant *Klebsiella pneumoniae* (MDR-KP) strains have increased worldwide. In addition, biofilm production by these resistant isolates has confronted clinicians with higher treatment failure and infection recurrence. Given the paucity of new agents and limited data on combination therapy for MDR-KPs, the present study sought to evaluate the in vitro activity of several antibiotic combinations against planktonic and biofilm MDR-KPs isolated from patients with VAP.

**Results:**

All 10 carbapenem-resistant *Klebsiella pneumoniae* (CRKP) isolates demonstrated multidrug resistance against the tested antibiotics. At planktonic mode, combinations of colistin-meropenem and amoxicillin/clavulanate in combination with meropenem, colistin, or amikacin showed synergism against 60–70% isolates. On the other hand, in the biofilm state, colistin-based combinations exhibited synergism against 50–70% isolates and the most effective combination was colistin-amikacin with 70% synergy.

**Conclusions:**

The results revealed that combinations of amoxicillin/clavulanate with colistin, meropenem, or amikacin in the planktonic mode and colistin with amoxicillin/clavulanate, meropenem, or amikacin in the biofilm mode could effectively inhibit CRKP isolates, and thus could be further explored for the treatment of CRKPs.

**Supplementary Information:**

The online version contains supplementary material available at 10.1186/s12866-023-03039-w.

## Introduction

*Klebsiella pneumoniae (K. pneumoniae*) is one of the most common pathogens responsible for various fatal infections, such as ventilator-associated pneumonia (VAP), which is the most common nosocomial infection [1].

The rising number of cases of antibiotic-resistant *K. pneumoniae* infection is a prominent health problem worldwide, presenting as a clinically important bacterium causing VAP among patients in intensive care units (ICUs) [2–4]. In addition to antibiotic resistance, one of the causes of death in these types of infections is the biofilm formation. Biofilm formation in the endotracheal tube of ventilated patients has been suggested to play a major role in the development of VAP [2, 5]. Compared with planktonic *K. pneumoniae*, bacterial cells in biofilm exhibit increased resistance to antibiotics and host immune responses, which may lead to chronic and recurrent infection, or treatment failure [6].

Carbapenem-resistant *K. pneumoniae* (CR-KP) remains a significant challenge associated with high mortality rate, exacerbated by the significant increase in the worldwide prevalence rate [7, 8]. Because of rising antibiotic resistance and biofilm tolerance to both antimicrobial and host immunological responses, clinicians are confronted with higher mortality rates, especially in critically ill patients [9].

Restricted therapeutic options retain activity against CR-KP infections, such as polymyxins. Polymyxins including colistin are important treatment options [10]. After being mostly abandoned in the 1970s, has led to renewed interest in reviving these cationic antimicrobial peptides as a last-resort antimicrobials, mainly for use against several carbapenem-resistant bacterial isolates [11, 12]. With the rise in colistin consumption, the emergence of colistin- and carbapenem-resistant *K. pneumoniae* (CCR-KP) strains has been reported globally [13, 14]. Resistance emergence to “last-line” drugs, such as colistin in CR-KP creates a therapeutic challenge that threatens to return clinicians and patients to a “pre-antibiotic era.”

Finding new and effective antimicrobial agents may be the ultimate strategy to combat these resistant bacteria, but its development takes a lot of resources and time. Hence, there’s a demand for a substitute, more fruitful, and more durable approach to deal with the status. Presently, numerous scientists are working on various aspects to tackle drug-resistant pathogens involving antimicrobial peptides, drug repurposing, antivirulence drugs, vaccination, phage therapy, and antibiotic combination therapy.

Antibiotic combination therapy can expand the sweep of clinical treatment, quicken bacterial clearance, rejuvenate old drugs, reduce the toxicity of used antibiotics, ameliorate antibiotic resistance, and consequently provide the solution [12, 15–25]. Therefore, optimizing the use of current antimicrobials and strategies based on synergistic effects is necessary to battle these resistant bacteria [26, 27].

It has been reported that several antimicrobial combinations suggest synergistic activity in vitro studies, and also some clinical observational studies indicate the superiority of combination therapy over monotherapy in the treatment of severe infections caused by carbapenemase-producing Enterobacteriaceae [12, 28–30]. However, conflicting results have been observed between studies, which might be due to strain-dependent factors and different methodologies.

When two antibiotics are used simultaneously, three types of interactions may occur. First, the “antagonistic effect”, where the potency of the combination is less than that of any single agent. Second, the” indifferent effect”, in which the strength of an antibiotic combination is more or less equal to the vigor of the combination of either antibiotic alone; and third, a” synergistic effect,“ in which two antibiotics produce a stronger effect than the combined strengths of each antibiotic alone [31, 32]. Since many of antimicrobials are candidates for combination therapy, selection based on the fractional inhibitory concentration index (FICI) [33] and utilization of the checkerboard assay [34] have been reported. In this study, we aimed to compare the activities of dual combinations of colistin, meropenem, amikacin, and amoxicillin/clavulanate against planktonic and biofilm CR-KPs and CCR-KPs isolates harboring different β-lactamase genes from patients with VAP.

## Results

### Characteristics of bacterial isolates

All of 10 isolates were obtained from the hospital laboratory of adults hospitalized in ICU setup. All isolates in addition to being carbapenem-resistant were Extended-Spectrum Beta-Lactamases (ESBL) and Metallo-Beta-Lactamase (MBL) producers (Table 1). In 8 strains, *bla*_OXA−48_, *bla*_CTX-M_ and *bla*_SHV_ genes were present simultaneously, and *bla*_VIM−1_ was also detected in 5 of these 8 isolates.

Antibiotic resistance in *K. pneumoniae* isolates studied was remarkably high. 90% (9/10) of them were resistant to ceftriaxone, ampicillin/sulbactam, and cefepime. In addition, 80% (8/10) of them were resistant to temocillin, trimethoprim/sulfamethoxazole, ciprofloxacin, tobramycin, piperacillin/tazobactam, and gentamicin, as shown in Table 1.

**Table 1:** Phenotypic and genotypic pattern of antibiotic resistance of 10 *K. pneumoniae* isolates

Isolates	Beta-Lactamases genes	Antibiotic resistance pattern
**CR-KP**		
K.1	*bla*_SHV_, *bla*_TEM_, *bla*_OXA-48_, *bla*_CTX-M_, *bla*_VIM-1_	TEM, TZP, IMI, MEM, GM, SXT, CIP, CRO, SAM, FEP, TOB, AMC
K.2	*bla*_SHV_, *bla*_TEM_, *bla*_OXA-48_, *bla*_CTX-M_, *bla*_NDM-1_	TEM, IMI, MEM, GM, CIP, CRO, SAM, AK, FEP, TOB, AMC
K.3	*bla*_SHV_, *bla*_TEM_, *bla*_OXA-48_, *bla*_CTX-M_	TEM, TZP, IMI, MEM, GM, SXT, CIP, CRO, SAM, AK, FEP, AMC
K.4	*bla*_SHV_, *bla*_VIM-1_	TEM, TZP, IMI, MEM, GM, SXT, CIP, CRO, SAM, AMC
K.5	*bla*_SHV_, *bla*_IMP_, *bla*_TEM_, *bla*_OXA-48_, *bla*_CTX-M_, *bla*_NDM-1_, *bla*_VIM-1_	TEM, TZP, IMI, MEM, GM, SXT, CIP, CRO, SAM, AK, FEP, TOB, AMC
**CCR-KP**		
K.6	*bla*_SHV_, *bla*_TEM_, *bla*_OXA-48_, *bla*_NDM-1_	IMI, MEM, CRO, SAM, FEP, TOB, AMC, COL
K.7	*bla*_SHV_, *bla*_TEM_, *bla*_OXA-48_, *bla*_CTX-M_	TZP, IMI, MEM, SXT, AK, FEP, TOB, AMC, COL
K.8	*bla*_SHV_, *bla*_TEM_, *bla*_OXA-48_, *bla*_CTX-M_, *bla*_VIM-1_	TEM, TZP, IMI, MEM, GM, SXT, CIP, CRO, SAM, AK, FEP, TOB, AMC, COL
K.9	*bla*_SHV_, *bla*_OXA-48_, *bla*_CTX-M_, *bla*_VIM-1_	TEM, TZP, IMI, MEM, GM, SXT, CIP, CRO, SAM, FEP, TOB, AMC, COL
K.10	*bla*_SHV_, *bla*_OXA-48_, *bla*_CTX-M_, *bla*_NDM-1_, *bla*_VIM-1_	TEM, TZP, IMI, MEM, GM, SXT, CIP, CRO, SAM, AK, FEP, TOB, AMC, COL

CR-KP: Carbapenem-resistant *Klebsiella pneumoniae*; CCR-KP: Colistin- and carbapenem-resistant *Klebsiella pneumoniae*:

TEM: temocillin; TZP: piperacillin/tazobactam; IMI: imipenem; MEM: meropenem; GM: gentamicin; SXT: trimethoprim/sulfamethoxazole; CIP: ciprofloxacin; CRO: ceftriaxone; SAM: ampicillin/sulbactam; AK: amikacin; FEP: cefepime; TOB: tobramycin; AMC: amoxicillin clavulanate; COL: colistin.

Minimum Inhibitory Concentration (MIC) ranges were 0.5–32 mg/mL, 4–256 mg/mL, 64/32-4096/2048 mg/mL, and 8–1024 mg/mL for colistin, meropenem, amoxicillin/clavulanate, and amikacin, respectively (Table 2). All isolates were resistant to meropenem and amoxicillin/clavulanate. 60% (6/10) and 50% (5/10) of isolates were resistant to amikacin and colistin, respectively. Three strains (30%) were resistant to all four antibiotics. Antimicrobial resistance patterns of the studied isolates are shown in Table 2.

On average, Minimum Biofilm Eradication Concentration (MBEC) was 5, 7, and 11-fold higher than MIC for colistin, amikacin, and meropenem, respectively. The MBEC of amoxicillin/clavulanate was greater than the concentration range investigated and reported as > 4096/2048 for all ten isolates (Table 2).

**Table 2:** MIC (mg/L) and MBEC (mg/L) antibiotics for 10 *K. pneumoniae* isolates

Clinical Isolate	Colistin		Meropenem		Amoxicillin/clavulanate		Amikacin	
	MIC	MBEC	MIC	MBEC	MIC	MBEC	MIC	MBEC
K.1	0.5	16	16	128	1024/512	> 4096/2048	32	128
K.2	1	16	16	256	1024/512	> 4096/2048	1024	4096
K.3	2	32	32	128	1024/512	> 4096/2048	256	2048
K.4	1	16	64	256	64/32	> 4096/2048	16	1024
K.5	2	16	256	4096	2048/1024	> 4096/2048	1024	8192
K.6	4	8	4	16	512/256	> 4096/2048	8	64
K.7	32	256	16	512	1024/512	> 4096/2048	64	1024
K.8	32	128	32	256	2048/1024	> 4096/2048	128	2048
K.9	32	64	32	64	512/256	> 4096/2048	32	512
K.10	16	64	256	2048	4096/2048	> 4096/2048	256	1024

### Checkerboard studies in planktonic mode

As shown in Table 3, in most cases the MIC was reduced in the combination mode compared to the single mode. Among the most obvious reductions, we can point out the 2-128-fold decrease in MIC of amikacin in combination with amoxicillin/clavulanate, the 4-32-fold decrease in MIC of amoxicillin/clavulanate in combination with colistin, and the 2-64-fold decrease in MIC of meropenem in combination with colistin.

**Fig. 1:**
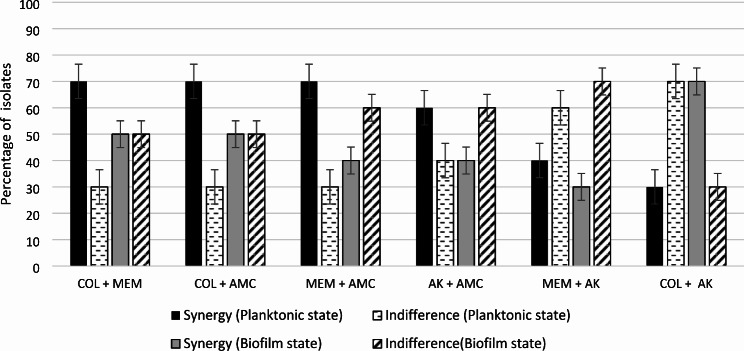
Distribution of FICI values ± SD for the combinations against *K. pneumoniae* isolates in planktonic and biofilm states. (COL: colistin; MEM: meropenem; AMC: amoxicillin clavulanate; AK: amikacin.)

Regarding the synergism studies on planktonic bacteria, meropenem-colistin combination, and amoxicillin/clavulanate combined with meropenem or colistin produced a synergistic activity against 7 of the 10 isolates (70%), while 3 isolates showed indifference results (30%) (Fig. 1). Amikacin combined with amoxicillin/clavulanate, meropenem, and colistin were synergistic against 60% (6/10), 40% (4/10), and 30% (3/10) of the isolates, respectively, other combinational effects were indifferent according to the FICIs (Fig. 1). Although colistin combined with amikacin showed indifference effect to most strains but this combination increased sensitivity to amikacin from 40 to 80% (Table 3). There was no significant difference in FICI values between CR-KP and CCR-KP isolates in the planktonic state.

In-vitro synergism results showed that synergy could be observed even though the isolate was resistant to one or both of the antibiotics in combination. In addition, the number of sensitive strains to antibiotics increased in all these studied combinations (Table 3), and no antagonism interaction was observed in planktonic mode.

**Table 3:** Effects of antibiotic combinations on 10 *K. pneumoniae* strains in planktonic state

Clinical Isolate	COL/ MEM		COL/ AMC		MEM / AMC		AK/COL		AK/ MEM		AK / AMC		
	MIC	FICI	MIC	FICI	MIC	FICI	MIC	FICI	MIC	FICI	MIC	FICI	
K.1	0.12/2	0.37 (S)	0.25/(256/128)	0.75 (I)	8/(512/256)	1.00 (I)	4/0.5	1.13 (I)	16/2	0.63 (I)	4/(128/64)	0.25 (S)	
K.2	0.5/4	0.75 (I)	0.25/(64/32)	0.38 (S)	4/(512/256)	0.75 (I)	512/0.03	0.53 (I)	64/8	0.56 (I)	512/(256/128)	0.75 (I)	
K.3	0.12/4	0.19 (S)	0.5/(64/32)	0.38 (S)	1/(256/128)	0.28 (S)	64/0.06	0.28 (S)	128/16	1.00 (I)	64/(512/256)	0.75 (I)	
K.4	0.25/16	0.50 (S)	0.12/(8/4)	0.25 (S)	16/(2/1)	0.28 (S)	4/0.12	0.38 (S)	2/8	0.25 (S)	8/(8/4)	0.63 (I)	
K.5	0.5/4	0.27 (S)	1/(256/128)	0.63 (I)	64/(256/128)	0.38 (S)	32/1	0.53 (I)	1024/128	1.50 (I)	256/(128/64)	0.31 (S)	
K.6	1/0.5	0.38 (S)	1/(128/64)	0.50 (S)	1/(64/32)	0.38 (S)	4/0.5	0.63 (I)	1/0.5	0.25 (S)	2/(128/64)	0.50 (S)	
K.7	16/16	1.50 (I)	8/(32/16)	0.28 (S)	8/(256/128)	0.75 (I)	16/16	0.75 (I)	4/4	0.31 (S)	1/(256/128)	0.27 (S)	
K.8	8/16	0.75 (I)	4/(64/32)	0.16 (S)	4/(512/256)	0.38 (S)	32/16	0.75 (I)	64/8	0.75 (I)	64/(512/256)	0.75 (I)	
K.9	1/4	0.16 (S)	16/(16/8)	0.53 (I)	4/(128/64)	0.38 (S)	2/16	0.56 (I)	16/4	0.75 (I)	0.25/(128/64)	0.26 (S)	
K.10	4/32	0.38 (S)	2/(1024/512)	0.50 (S)	16/(512/256)	0.19 (S)	32/4	0.38 (S)	32/32	0.25 (S)	32/(1024/512)	0.38 (S)	

COL: colistin; MEM: meropenem; AMC: amoxicillin clavulanate; AK: amikacin. MIC, minimum inhibitory concentration that expressed in mg/L. S, synergy; I, indifferent.

### Checkerboard studies in biofilm mode

As shown in Table 4, in most cases, the MBEC was reduced in the combination mode compared to the single mode. Among the most obvious reductions, decline of at least 2–64 fold in MBEC of amoxicillin/clavulanate in combination with colistin and the 2-32-fold decrease in MBEC of meropenem and amikacin in combination with colistin could be mentioned.

Colistin-amikacin combination produced a synergistic activity against 70% (7/10) isolates, while 30% (3/10) isolates showed indifference results. Colistin combined with meropenem, or amoxicillin/clavulanate exhibited a synergistic activity against half of the isolates (50%), while the other half isolates produced indifference results (50%) (Fig. 1). In biofilm mode, amoxicillin/clavulanate combined with meropenem, or amikacin exhibited indifference effect against 6 of the 10 isolates, and just 4 isolates showed synergistic activity (Fig. 1). The lowest synergism was related to meropenem-amikacin combination (3 of the 10 isolates). There was no significant difference in FICI values between CR-KP and CCR-KP isolates in the biofilm state, and none of the isolates showed antagonism effect for the studied combinations in the biofilm mode.

**Table 4:** Effects of combinations on 10 *K. pneumoniae* strains in biofilm state

Clinical Isolate	COL / MEM		COL / AMC		MEM / AMC		AK / COL		AK / MEM		AK / AMC		
	MBEC	FICI	MBEC	FICI	MBEC	FICI	MBEC	FICI	MBEC	FICI	MBEC	FICI	
K.1	8/4	0.53 (I)	8/(2048/1024)	0.75 (I)	32/(4096/2048)	0.75 (I)	32/4	0.50 (S)	32/64	0.75 (I)	32/(4096/2048)	0.75 (I)	
K.2	4/32	0.38 (S)	2/(512/256)	0.19 (S)	64/(512/256)	0.31 (S)	1024/8	0.75 (I)	1024/64	0.50 (S)	1024/(256/128)	0.28 (S)	
K.3	8/32	0.50 (S)	8/(128/64)	0.27 (S)	32/(256/128)	0.28 (S)	1024/16	1.00 (I)	1024/64	1.00 (I)	1024/(4096/2048)	1.00 (I)	
K.4	4/128	0.75 (I)	4/(256/128)	0.28 (S)	16/(1024/512)	0.19 (S)	128/2	0.25 (S)	512/64	0.75 (I)	512/(1024/512)	0.63 (I)	
K.5	4/256	0.31 (S)	1/(2048/1024)	0.31 (S)	256/(4096/2048)	0.56 (I)	2048/4	0.50 (S)	4096/2048	1.00 (I)	2048/(2048/1024)	0.50 (S)	
K.6	4/4	0.75 (I)	4/(256/128)	0.53 (I)	8/(2048/1024)	0.75 (I)	8/2	0.38 (S)	8/2	0.25 (S)	32/(2048/1024)	0.75 (I)	
K.7	64/256	0.75 (I)	64/(4096/2048)	0.75 (I)	256/(256/128)	0.53 (I)	256/128	0.75 (I)	512/128	0.75 (I)	64/(512/256)	0.13 (S)	
K.8	32/16	0.31 (S)	32/(4096/2048)	0.75 (I)	32/(512/256)	0.19 (S)	128/32	0.31 (S)	256/32	0.25 (S)	128/(512/256)	0.13 (S)	
K.9	8/64	1.13 (I)	16/(128/64)	0.27 (S)	64/(2048/1024)	1.25 (I)	32/16	0.31 (S)	128/32	0.75 (I)	256/(256/128)	0.53 (I)	
K.10	16/256	0.38 (S)	32/(1024/512)	0.63 (I)	256/(4096/2048)	0.63 (I)	256/16	0.50 (S)	256/1024	0.75 (I)	256/(4096/2048)	0.75 (I)	

COL: colistin; MEM: meropenem; AMC: amoxicillin clavulanate; AK: amikacin. MBEC, minimum biofilm eradication concentration that expressed in mg/L. S, synergy; I, indifferent.

## Discussion

The rapid spread of multiple-drug resistant CR-KP isolates threaten the continued usage of antibiotics chemotherapy and places an enormous burden on global healthcare systems. Furthermore, the search, finding, and development of new antibiotics is extremely time-consuming with no novel discovery over the last 30 years. This shortage of effective therapeutic agents has encouraged the use of combination therapy of existing agents for synergistic effects against drug-resistant isolates [35–38]. In addition combination therapy is needed not only to inhibit microbial proliferation through a multi-target approach but also to prevent the emergence of the resistance during the treatment.

In this study, we applied the checkerboard techniques for the evaluation of the efficacies of colistin-meropenem, colistin-amoxicillin/clavulanate, meropenem- amoxicillin/clavulanate, amikacin-colistin, amikacin–meropenem and amikacin- amoxicillin/clavulanate combinations against selected CR-Kp tracheal isolates in planktonic and biofilm modes, and the obtained results classified based on FICI parameter (Tables 3 and 4).

Antibiotic combinations demonstrated 2-to 32-fold reduction in MIC of colistin and amoxicillin/clavulanate, 2-to 64-fold reduction in MIC of meropenem, and 2-to 128-fold reduction in MIC of amikacin.

A summary of the results indicates that in the planktonic mode meropenem-colistin and amoxicillin/clavulanate with meropenem, or colistin combinations were synergistic against 70% (7 of 10 isolates). Amikacin with amoxicillin/clavulanate exhibited synergism against 60% (6 of 10 isolates), while combination of amikacin with meropenem and colistin displayed 40% (4 in 10 isolates), and 30% (3 of 10 isolates) synergism, respectively.

In our study, similar to several previous studies, the high synergism of colistin-meropenem combination was illustrated [35, 39]. Goel et al. detected the higher synergism by the Etest (82%), checkerboard (88%) and time-kill (78%) methods for this combination compared to us [35, 39]. Conducted studies by Dhandapani et al. and Daoud et al. with the combination of meropenem and colistin showed 56% and 54% synergy, respectively [40, 41], which is less than our study results. A study by V.L. Minh et al. with the combination of colistin with various antibiotics on carbapenem resistant isolates of Acinetobacter spp showed 68% synergy which is close to our results. The present experience showed high synergism for amoxicillin/clavulanate based combinations. The use of amoxicillin/clavulanate in combinations caused a 20% increase in susceptible to second antibiotic. In the insights of these results, despite the high MIC of amoxicillin/clavulanate, this antibiotic in combination with meropenem, colistin, and amikacin exhibited a synergy effect comparable to colistin-meropenem; therefore, amoxicillin/clavulanate combined with colistin, meropenem, or amikacin could be suggested as a treatment option against CR-KPs in order to further investigations.

Our in vitro results on combination activity proposed different behaviors for bacteria in a planktonic and biofilm states.

In the biofilm mode, combinations of colistin with amikacin was synergistic against 70% (7 of 10 isolates), and colistin combined with meropenem or amoxicillin/clavulanate exhibited a synergistic activity against 50% (5 of 10 isolates). The results revealed that colistin in combination with three other antibiotics could effectively inhibit the biofilm of *K. pneumoniae* isolates. Furthermore, colistin showed the least increase in MBEC compared to MIC (on average, about 5-fold) and was the most successful antibiotic in reducing adjuvant antibiotic’s MBEC. As a result, colistin, both alone and in combination, was the most effective antibiotic in our study at the biofilm state. In another study, Wang et al. were assessed the activity of colistin-amikacin combination against *Pseudomonas aeruginosa* isolates both in vitro and in vivo by counting the live bacteria in biofilm and an animal biofilm infection model. They reported that colistin-amikacin combination could shorten the eradication time of biofilm than monotherapy [42].

The mechanism of synergy in colistin-based combinations in the biofilm state is likely to be linked to colistin-mediated changes in biofilm structure including reduction of cell density and swelling of biofilm matrix [43] which allowed entry of colistin and second antibiotic into the biofilm matrix. Furthermore, to weaken the biofilm, colistin affects the cells embedded in the inner layers of the biofilm structure, which are metabolically inactive [44].

In our study, effective antibiotic combinations in the planktonic state were different from the biofilm state, consequently it can be concluded that the effective combinations in acute infections are probably different from chronic infections.

No significant correlation was detected between the resistance genotypic pattern and the interaction of antibiotics in the combination, and therefore, the observed synergy could not be considered dependent on the molecular pattern of beta-lactamase resistance.

Furthermore, there was no significant difference between the FICI values in the two CR-KP and CCR-KP groups in the planktonic and biofilm states. Consequently, it seems that the sensitivity or resistance to colistin had not a predictable effect on the results of using the studied antibiotic combinations against MDR-KP isolates.

## Conclusions

In conclusion, we demonstrated that amoxicillin/clavulanate combined with colistin, meropenem, or amikacin effectively inhibit CRKPs in planktonic mode and colistin-based antibiotic combinations, especially in combination with aminoglycosides such as amikacin are effective at CRKPs biofilm. Therefore, we suggest these combinations be further explored in the future study for the treatment of CRKPs.

There are a few limitations in this study. Firstly, while our in vitro tests showed antimicrobial activity of studied antibiotic combinations, the findings from this study need to be validated in vivo and in clinical trials. Secondly, we only tested 10 CRKP isolates from a Healthcare Center with combination treatments which calls for further evaluation against a wider group of CRKP isolates.

## Materials and methods

### Bacterial isolates

From a collection of *K. pneumoniae* isolates, 10 MDR and strong biofilm producer isolates (five CR-KPs and five CCR-KPs), which were obtained from tracheal aspirate specimens of patients with VAP in a tertiary hospital clinical laboratory, selected for the present study. The phenotypic and genotypic resistance profile of studied isolates are listed in Table 1. The primers used in polymerase chain reaction (PCR) tests in order to detect the resistance genes are listed in Supplementary file 1.

### Determination of MIC

MIC, defined as the lowest antibiotic concentration (µg/ml) required to stop bacterial growth was determined for meropenem, colistin, amoxicillin/clavulanate and amikacin by using the Clinical Laboratory Standards Institute (CLSI) [45].

The MIC results were interpreted per CLSI criteria except for colistin results, which were analyzed based on the European Committee on Antimicrobial Susceptibility Testing (EUCAST) breakpoint recommendations [46]. *Escherichia coli* ATCC 25,922, *Staphylococcus aureus* ATCC 29,213, *Enterococcus faecalis* ATCC 29,212, and *K. pneumoniae* ATCC700603 were used as quality control.

### Determination of MBEC

The MBEC was determined based on the modified previous protocol [47]. Biofilms were grown on the surface of flat-bottomed 96-well microtiter plates at 37 °C for 24 h, and then wells were washed carefully twice with sterile PBS. Biofilms were subsequently incubated with 2-fold dilutions of colistin, meropenem, amoxicillin/clavulanate, and amikacin at concentrations ranging from 0.5 to 512 time MIC for 24 h. Drug-free biofilms served as untreated controls.

Metabolic activity was evaluated through the (3-[4,5-dimethylthiazol-2-yl]-2,5-diphenyl tetrazolium bromide) MTT (DNAbiotech Co) reduction assay, and the concentration producing a ≥ 50% biofilm damage compared to untreated controls was defined as MBEC. The percent biofilm damage was calculated according to the formula that was described by Geladari et al. [48]. Whenever the highest antibiotic concentration failed to achieve a 50% reduction in metabolic activity, the MBEC was defined as a concentration higher than the highest concentration tested.

### Checkerboard experiments in planktonic and biofilm models

The two-dimensional checkerboard microdilution method was used to evaluate synergism between antibiotics in colistin-meropenem, colistin-amoxicillin/clavulanate, meropenem- amoxicillin/clavulanate, amikacin-colistin, amikacin–meropenem and amikacin- amoxicillin/clavulanate combinations on *K. pneumoniae* isolates in planktonic and biofilm modes [49].

In planktonic mode, the antibiotics were diluted in Mueller Hinton Broth (MHB) to achieve a starting concentration equivalent to 2 time MIC. First antibiotic was serially diluted along the x-axis (the abscissa) of the 96-well microtiter plate, whereas second compound was serially diluted along the y-axis (the ordinate) to form a matrix. Antibiotics concentrations ranged from 0.031 to 2 time MIC for each isolate was studied. Bacteria equivalent to a McFarland standard of 0.5 were prepared in normal saline. The bacteria were then diluted in MHB to achieve a starting cell density of 1 time ${10}^{5}$ CFU/ml. The bacterial suspension was transferred to the microtiter plate. Wells containing MHB with or without bacterial cells were used as positive or negative controls, respectively. The microtiter plate was incubated for 24 h at 37 °C, and record the MIC of the single drug and the dual combination.

The activity of the combinations against bacterial biofilm was evaluated as follows:200 µL of antibiotic dilutions alone or in combination were added to each well where a 24 h biofilm had formed, and then the plates were incubated at 37 °C for 24 h. Antibiotic concentrations ranged from 0.031 to 2 time MBEC of each isolate was studied. MBEC was evaluated through the MTT reduction assay as described above. Experiments were repeated two times, and the results were concordant.

The effects of the antimicrobial combinations were defined according to the FICI as follows: FICI ≤ 0.5, synergism; 0.5 < FICI ≤ 4, indifferent; or FICI > 4, antagonistic [49].

### Electronic supplementary material

Below is the link to the electronic supplementary material.

Supplementary Material 1

## Data Availability

All data generated or analyzed during this study are included here and are available from the corresponding author on reasonable request.

## Abbreviations

MDR-KP: Multidrug-Resistant *Klebsiella pneumoniae*
*K. pneumoniae*: *Klebsiella pneumoniae*
VAP: Ventilator-Associated Pneumonia
ICUs: Intensive Care Units
CR-KP: Carbapenem- Resistant *K. pneumoniae*
CCR-KP: Colistin- and Carbapenem-Resistant *K. pneumoniae*
FICI: Fractional Inhibitory Concentration Index
ESBL: Extended-Spectrum Beta-Lactamases
MBL: Metallo-Beta-Lactamase
MBEC: Minimum Biofilm Eradication Concentration
MIC: Minimum Inhibitory Concentration
CLSI: Clinical Laboratory Standards Institute
EUCAST: European Committee on Antimicrobial Susceptibility Testing
MHB: Mueller Hinton Broth

## References

[1] Kalil AC, Metersky ML, Klompas M, Muscedere J, Sweeney DA, Palmer LB (2016). Management of adults with hospital-acquired and ventilator-associated pneumonia: 2016 clinical practice guidelines by the infectious Diseases Society of America and the american thoracic society. Clin Infect Dis.

[2] Moradi M, Nili F, Nayeri F, Amini E, Esmaeilnia T. Study of characteristics, risk factors and outcome for Ventilator Associated Pneumonia in neonatal intensive care unit patient. Tehran Univ Med J. 2013;71(6).

[3] Kalanuria AA, Mirski M, Ziai W (2014). Ventilator-associated pneumonia in the ICU. Annual Update in Intensive Care and Emergency Medicine.

[4] Selina F, Talha KA, Islam A, Hasan Z, Hyder M, Selvapandian S (2009). Organisms associated with ventilator associated pneumonia (VAP) in intensive care units (ICU). J Bangladesh Soc Anaesthesiologists.

[5] Gil-Perotin S, Ramirez P, Marti V, Sahuquillo JM, Gonzalez E, Calleja I (2012). Implications of endotracheal tube biofilm in ventilator-associated pneumonia response: a state of concept. Crit Care.

[6] Donlan RM, Costerton JW (2002). Biofilms: survival mechanisms of clinically relevant microorganisms. Clin Microbiol Rev.

[7] Munoz-Price LS, Poirel L, Bonomo RA, Schwaber MJ, Daikos GL, Cormican M (2013). Clinical epidemiology of the global expansion of Klebsiella pneumoniae carbapenemases. Lancet Infect Dis.

[8] van Duin D, Perez F, Rudin SD, Cober E, Hanrahan J, Ziegler J (2014). Surveillance of carbapenem-resistant Klebsiella pneumoniae: tracking molecular epidemiology and outcomes through a regional network. Antimicrob Agents Chemother.

[9] Gominet M, Compain F, Beloin C, Lebeaux D (2017). Central venous catheters and biofilms: where do we stand in 2017?. Apmis.

[10] van Duin D, Kaye KS, Neuner EA, Bonomo RA (2013). Carbapenem-resistant Enterobacteriaceae: a review of treatment and outcomes. Diagn Microbiol Infect Dis.

[11] Qureshi ZA, Hittle LE, O’Hara JA, Rivera JI, Syed A, Shields RK (2015). Colistin-resistant Acinetobacter baumannii: beyond carbapenem resistance. Clin Infect Dis.

[12] Tumbarello M, Viale P, Viscoli C, Trecarichi EM, Tumietto F, Marchese A (2012). Predictors of mortality in bloodstream infections caused by Klebsiella pneumoniae carbapenemase–producing K. pneumoniae: importance of combination therapy. Clin Infect Dis.

[13] Van Duin D, Doi Y, Commentary (2015). Outbreak of Colistin-Resistant, carbapenemase-producing Klebsiella pneumoniae: are we at the end of the Road?. J Clin Microbiol.

[14] Gelbicova T, Kolackova I, Krutova M, Karpiskova R (2020). The emergence of mcr-1-mediated colistin-resistant Escherichia coli and Klebsiella pneumoniae in domestic and imported turkey meat in the Czech Republic 2017–2018. Folia Microbiol.

[15] Doern CD (2014). When does 2 plus 2 equal 5? A review of antimicrobial synergy testing. J Clin Microbiol.

[16] Xu X, Xu L, Yuan G, Wang Y, Qu Y, Zhou M (2018). Synergistic combination of two antimicrobial agents closing each other’s mutant selection windows to prevent antimicrobial resistance. Sci Rep.

[17] Bollenbach T (2015). Antimicrobial interactions: mechanisms and implications for drug discovery and resistance evolution. Curr Opin Microbiol.

[18] Vidaillac C, Benichou L, Duval RE (2012). In vitro synergy of colistin combinations against colistin-resistant Acinetobacter baumannii, Pseudomonas aeruginosa, and Klebsiella pneumoniae isolates. Antimicrob Agents Chemother.

[19] Paul M, Daikos GL, Durante-Mangoni E, Yahav D, Carmeli Y, Benattar YD (2018). Colistin alone versus colistin plus meropenem for treatment of severe infections caused by carbapenem-resistant Gram-negative bacteria: an open-label, randomised controlled trial. Lancet Infect Dis.

[20] Mitchison DA. Prevention of drug resistance by combined drug treatment of tuberculosis. Antibiotic Resist. 2012:87–98. 10.1007/978-3-642-28951-4_6 23090597

[21] Kerantzas CA, Jacobs WR (2017). Origins of combination therapy for tuberculosis: lessons for future antimicrobial development and application. MBio.

[22] Caballero J, Rello J (2011). Combination antibiotic therapy for community-acquired pneumonia. Ann Intensiv Care.

[23] Zhou A, Kang TM, Yuan J, Beppler C, Nguyen C, Mao Z (2015). Synergistic interactions of vancomycin with different antibiotics against Escherichia coli: trimethoprim and nitrofurantoin display strong synergies with vancomycin against wild-type E. coli. Antimicrob Agents Chemother.

[24] Drusano GL, Neely M, Van Guilder M, Schumitzky A, Brown D, Fikes S (2014). Analysis of combination drug therapy to develop regimens with shortened duration of treatment for tuberculosis. PLoS ONE.

[25] Eliopoulos GM, Moellering RC (1982). Antibiotic synergism and antimicrobial combinations in clinical infections George. Rev Infect Dis.

[26] Chi H, Holo H (2018). Synergistic antimicrobial activity between the broad spectrum bacteriocin garvicin KS and nisin, farnesol and polymyxin B against gram-positive and gram-negative bacteria. Curr Microbiol.

[27] Rice LB (2009). The clinical consequences of antimicrobial resistance. Curr Opin Microbiol.

[28] Tzouvelekis L, Markogiannakis A, Psichogiou M, Tassios P, Daikos G (2012). Carbapenemases in Klebsiella pneumoniae and other Enterobacteriaceae: an evolving crisis of global dimensions. Clin Microbiol Rev.

[29] Qureshi ZA, Paterson DL, Potoski BA, Kilayko MC, Sandovsky G, Sordillo E (2012). Treatment outcome of bacteremia due to KPC-producing Klebsiella pneumoniae: superiority of combination antimicrobial regimens. Antimicrob Agents Chemother.

[30] Kontopidou F, Giamarellou H, Katerelos P, Maragos A, Kioumis I, Trikka-Graphakos E (2014). Infections caused by carbapenem-resistant Klebsiella pneumoniae among patients in intensive care units in Greece: a multi-centre study on clinical outcome and therapeutic options. Clin Microbiol Infect.

[31] Tallarida RJ (2011). Quantitative methods for assessing drug synergism. Genes & cancer.

[32] Gunnison J, Shevky M, Bruff J, Coleman V, Jawetz E (1953). Studies on antibiotic synergism and antagonism: the effect in vitro of combinations of antibiotics on bacteria of varying resistance to single antibiotics. J Bacteriol.

[33] Chin N-X, Neu HC (1983). Synergy of azlocillin with aminoglycosides. J Antimicrob Chemother.

[34] Tateda K, Ishii Y, Matsumoto T, Yamaguchi K (2006). Break-point checkerboard plate’for screening of appropriate antibiotic combinations against multidrug-resistant Pseudomonas aeruginosa. Scand J Infect Dis.

[35] Yu L, Zhang J, Fu Y, Zhao Y, Wang Y, Zhao J (2019). Synergetic effects of combined treatment of colistin with meropenem or amikacin on carbapenem-resistant Klebsiella pneumoniae in vitro. Front Cell Infect Microbiol.

[36] Kilic U, Koroglu M, Olmez M, Altindis M (2020). Investigation of the in vitro effectiveness of aztreonam/avibactam, colistin/apramycin, and meropenem/apramycin combinations against carbapenemase-producing, extensively drug-resistant Klebsiella pneumoniae strains. Microb Drug Resist.

[37] Erdem F, Abulaila A, Aktas Z, Oncul O (2020). In vitro evaluation of double carbapenem and colistin combinations against OXA-48, NDM carbapenemase-producing colistin-resistant Klebsiella pneumoniae strains. Antimicrob Resist Infect Control.

[38] Petrosillo N, Giannella M, Lewis R, Viale P (2013). Treatment of carbapenem-resistant Klebsiella pneumoniae: the state of the art. Expert Rev anti-infective Therapy.

[39] Goel A, Gupta V, Singhal L, Palta S, Chander J (2021). In vitro evaluation of antibiotic synergy for carbapenem-resistant Klebsiella pneumoniae clinical isolates. Indian J Med Res.

[40] Dhandapani S, Sistla S, Gunalan A, Manoharan M, Sugumar M, Sastry AS (2021). In-vitro synergistic activity of colistin and meropenem against clinical isolates of carbapenem resistant E. coli and Klebsiella pneumoniae by checkerboard method. Ind J Med Microbiol.

[41] Daoud Z, Mansour N, Masri K. Synergistic combination of carbapenems and colistin against P. aeruginosa and A. baumannii. Open J Med Microbiol. 2013;2013.

[42] Wang Y, Li C, Wang J, Bai N, Zhang H, Chi Y (2022). The efficacy of Colistin combined with amikacin or levofloxacin against Pseudomonas aeruginosa Biofilm infection. Microbiol Spectr.

[43] Klinger-Strobel M, Stein C, Forstner C, Makarewicz O, Pletz MW (2017). Effects of colistin on biofilm matrices of Escherichia coli and Staphylococcus aureus. Int J Antimicrob Agents.

[44] Li J, Nation RL, Kaye KS. Polymyxin antibiotics: from laboratory bench to bedside. Springer; 2019.

[45] Weinstein MP, Limbago B, Patel J, Mathers A, Campeau S, Mazzulli T (2018). M100 performance standards for antimicrobial susceptibility testing. CLSI.

[46] Kahlmeter G, Brown D, Goldstein F, MacGowan A, Mouton J, Odenholt I, et al. European Committee on Antimicrobial susceptibility testing (EUCAST) technical notes on antimicrobial susceptibility testing. Wiley Online Library; 2006. pp. 501–3. 10.1111/j.1469-0691.2006.01454.x 16700696

[47] Bardbari AM, Arabestani MR, Karami M, Keramat F, Aghazadeh H, Alikhani MY (2018). Highly synergistic activity of melittin with imipenem and colistin in biofilm inhibition against multidrug-resistant strong biofilm producer strains of Acinetobacter baumannii. Eur J Clin Microbiol Infect Dis.

[48] Geladari A, Simitsopoulou M, Antachopoulos C, Roilides E (2019). Dose-dependent synergistic interactions of colistin with rifampin, meropenem, and tigecycline against carbapenem-resistant Klebsiella pneumoniae biofilms. Antimicrob Agents Chemother.

[49] Leber AL. Clinical microbiology procedures handbook. John Wiley & Sons; 2020.

