# Supplementary material for: In Vitro antibiotic combinations of Colistin, Meropenem, Amikacin, and Amoxicillin/clavulanate against multidrug-resistant Klebsiella pneumonia isolated from patients with ventilator-associated pneumonia
Source: BMC Microbiol. 2023 Oct 20;23:298. doi: 10.1186/s12866-023-03039-w (PMC10588070; doi:10.1186/s12866-023-03039-w)
Supplement: Supplementary file 1 — Supplementary Material 1 [file 12866_2023_3039_MOESM1_ESM.docx]

| Gene | **Primer Sequence (5’ to 3’)** | **Product size (bp)** | **Annealing T (^°^C)** | **Ref** |
| --- | --- | --- | --- | --- |
| **ESBL** |  |  |  |  |
| *bla*_SHV_ | F- AGCCGCTTGAGCAAATTAAAC  R- ATCCCGCAGATAAATCACCAC | 712 | 55,6 | (50) |
| *bla*_TEM_ | F- TGCGGTATTATCCCGTGTTG  R- TCGTCGTTTGGTATGGCTTC | 296 | 63 | (51) |
| *bla*_CTX-M_ | F- CGCTTTGCGATGTGA  R- ACCGCGATATCGTTG | 550 | 60 | (52) |
| **MBL** |  |  |  |  |
| *bla*_IMP_ | F- CTACCGCAGCAGAGTCTTTGC  R- ACAACCAGTTTTGCCTTACC | 587 | 55 | (53) |
| *bla*_OXA-48_ | F- GCGTGGTTAAGGATGAACAC  R- CATCAAGTTCAACCCAACCG | 438 | 55 | (54) |
| *bla*_NDM-1_ | F- GAA GCT GAG CAC CGC ATT AG  R- GGG CCG TAT GAG TGA TTG C | 621 | 59 | (55) |
| *bla*_VIM-1_ | F- AGTGGTGAGTATCCGACAG  R- ATGAAAGTGCGTGGAGAC | 261 | 52 | (56) |

Suplementary file 1. Oligonucleotide sequences used in this study
